# Late mismatch negativity of lexical tone at age 8 predicts Chinese children’s reading ability at age 10

**DOI:** 10.3389/fpsyg.2022.989186

**Published:** 2022-10-21

**Authors:** Han Wu, Yixiao Zhang

**Affiliations:** ^1^Institute on Education Policy and Evaluation of International Students, Beijing Language and Culture University, Beijing, China; ^2^Faculty of Electronic Information and Electrical Engineering, School of Biomedical Engineering, Dalian University of Technology, Dalian, China

**Keywords:** categorical perception, lexical tone, reading ability, late MMN, children

## Abstract

**Background:**

Deficits in phonological processing are commonly reported in dyslexia but longitudinal evidence that poor speech perception compromises reading is scant. This 2-year longitudinal ERP study investigates changes in pre-attentive auditory processing that underlies categorical perception of mandarin lexical tones during the years children learn to read fluently. The main purpose of the present study was to explore the development of lexical tone categorical perception to see if it can predict children’s reading ability.

**Methods:**

Both behavioral and electrophysiological measures were taken in this study. Auditory event-related potentials were collected with a passive listening oddball paradigm. Using a stimulus continuum spanning from one lexical tone category exemplar to another, we identified a between-category and a within-category tone deviant that were acoustically equidistant from a standard stimulus. The standard stimulus occurred on 80% of trials, and one of two deviants (between-category or within-category) equiprobably on the remaining trials. 8-year-old Mandarin speakers participated in both an initial ERP oddball paradigm and returned for a 2-year follow-up.

**Results:**

The between-category MMN and within-category MMN significantly correlate with each other at age 8 (*p* = 0.001) but not at age 10. The between-category MMN at age 8 can predict children’s ability at age 10 (*p* = 0.03) but the within-category cannot.

**Conclusion:**

The categorical perception of lexical tone is still developing from age 8 to age 10. The behavioral and electrophysiological results demonstrate that categorical perception of lexical tone at age 8 predicts children’s reading ability at age 10.

## Introduction

Reading is a complex process that involves a wide range of cognitive abilities. The exploration of reading-related cognitive abilities has been an important issue of great interest to researchers. The relationship between categorical perception, phonological awareness, and reading has been widely concerned by researchers (Meng et al., 2005; McBride-Chang et al., 2008; Shu et al., 2008). Researchers initially explored these relationships through dyslexia to see whether they have problems with speech perception and phonological awareness. Some research found that individuals with dyslexia have problems with categorical perception (Joanisse et al., 2000; Franck et al., 2003; Liu et al., 2009), as evidenced by weaker discrimination of between-category differences and better discrimination of within-category differences compared to average readers (Werker and Tees, 1987; Serniclaes et al., 2001; Zhang et al., 2012). It is generally believed that poor categorization perception leads to fuzzy speech representation, which affects the development of reading ability (McBride-Chang, 1996; Zhang and McBride-Chang, 2010; Snowling et al., 2019).

Nevertheless, as the studies were conducted, inconsistent results were found. The research of O’Brien et al. found that categorical perception is less related to phonological awareness and more related to reading. Categorical perception may not act on reading through phonological awareness, but has a more direct relationship with reading (O'Brien et al., 2018). This suggests that the relationship between speech perception and phonological awareness as well as reading is very complex. Therefore, an in-depth investigation of this issue would need to be achieved through longitudinal studies that explore the predictive role of perceptual abilities on reading at critical stages of children’s reading development. In terms of behavioral task performance, 8 years old children already have the capacity for categorical perception (Hazan and Barrett, 2000), and 8 years of age is also an important turning point in the development of reading skills (Cheung et al., 2009), gradually becoming fluent readers. Therefore, exploring the predictive role of speech perception on phonological awareness as well as reading in 8 years old children would be able to fill the research gap and demonstrate the relationship between speech perception, phonological awareness, and reading.

Previous studies investigate categorical perception by Identification task and discrimination task; however, behavior tasks are not differentiated enough and are vulnerable to irrelevant factors such as motivation. Therefore, the use of passive electrophysiological methods is gaining popularity. As its paradigm is child-friendly, the mismatch negativity component has been established as one of the neural indexes of the categorical perception (Zhang et al., 2012). The auditory mismatch negativity (MMN) is a change-specific component of the auditory event-related brain potential (ERP) elicited through oddball paradigms irrespective of the subject’s direction of attention (Naatanen and Michie, 1979). The MMN can be elicited even in newborns and infants (Cheour et al., 1998; Cheng and Lee, 2018). Studies have demonstrated that MMN to speech sounds predict reading failure longitudinally. The larger the MMR (mismatch responses including both mismatch negativity and mismatch positivity) amplitude is, the better is reading and spelling performance (Schulte-Korne et al., 1998; Leppanen et al., 2002; Espy et al., 2004; Maurer et al., 2009; Leppänen et al., 2010; Van Zuijen et al., 2012; Hamalainen et al., 2013; Plakas et al., 2013; Schaadt et al., 2015). In children, the MMN usually peaks at 150–250 ms from change onset, followed by another negativity component peaking between 400 and 500 ms, which is named “late MMN” (Korpilahti and Lang, 1994; Korpilahti et al., 2001; Bishop and McArthur, 2004).

Late MMN mainly appeared in the study of young children, Bishop et al. suggest that it reflects the further processing of the salient features of the stimulus that are difficult to discriminate and the processes of conscious detection of a complex stimulus change (Korpilahti et al., 2001; Bishop et al., 2011; Turco et al., 2018). It is suggested to be associated with higher cognitive processes such as attention (Shestakova et al., 2003) or long-term memory (Zachau et al., 2005). Some researchers suggested that late MMN may reflect attention reorienting itself back to the original task (Yang et al., 2015). For a more comprehensive understanding of the auditory processing, it is important to focus on not only early MMN, but also late MMN (Choudhury et al., 2015).

The “late MMN” sometimes called the late discriminative negativity (LDN; Cheour et al., 2001; Naatanen et al., 2012; Fitzgerald and Todd, 2020), which is widely used in both the children’s and patients’ domains. Its amplitude, morphology, or degree of lateralization can be influenced by age or disease. For the children’s developmental domain, its amplitude tends to decrease with age (Cheour et al., 2001; Korpilahti et al., 2001; Linnavalli et al., 2018). For the patients’ domain, there are some important conclusions. For example: (a) Late MMN is a neurophysiological endophenotype for dyslexia (Neuhoff et al., 2012), and its amplitude is reduced in adults and children with dyslexia (Schulte-Korne et al., 2001; Hamalainen et al., 2008). For the children at familial dyslexia risk, its amplitude attenuated and its left lateralized was less (Maurer et al., 2003). Dyslexia’s auditory impairment reflected by late MMN may reflect a variation in the organization of the cortex rather than a developmental delay (Hommet et al., 2009). (b) Late MMN is also a neurophysiological endophenotype for SLI (specific language impairment). As the morphology of late MMN is different between SLI children and normal children (Korpilahti et al., 2001). (c) Late MMN is a neurophysiological endophenotype for cochlear implant children. For cochlear implant children’s neural activity of speech induced (Ahmadi et al., 2022). These studies all illustrate an important point: the late MMN is a good neural marker of auditory processing ability. In our study, we explored the relationship between-category perception and reading ability through late MMN evoked by between-category and within-category stimulus in the hope of seeing the predictive role of the late MMN as a neural marker of categorical perceptual abilities for reading ability.

Chinese is markedly different from alphabetic language. In alphabetic language, previous studies on categorization perception mainly focused on segmental information such as consonants and vowels. In Chinese, however, suprasegmental phonological processing (i.e., lexical tone perception) is a potential factor that accounts for reading difficulty in Chinese (Tong et al., 2018). Mandarin Chinese is a tonal language. Chinese syllables obligatorily carry tones, which are as critical as consonants and vowels. Gandour et al. (2003) demonstrated that tones were processed by the left hemisphere in Chinese listeners. In addition, the perception of tones has been shown to correlate with Chinese reading acquisition (Meng et al., 2005; McBride-Chang et al., 2008; Shu et al., 2008). Imprecise lexical tone perception is essential to account for reading learning difficulties in Mandarin-speaking children (Liu and Tsao, 2017). These findings suggest that lexical tone perception plays an important role in reading development in Chinese native speakers.

Better understanding of the neurobiological mechanisms in language development will lead to more effective educational and intervention strategies. Children could profit from additional help at the beginning of reading acquisition. If the goal is to understand the factors contributing to language-learning, it would be most informative to document the developmental progression as it unfolds. Maurer’s study in 2009 showed that late MMN in kindergarten can predict reading at grade 5. This prediction has an additional contribution based on behavioral scores (Maurer et al., 2009). We are particularly concerned about the speech perception of children from 8 to 10 years old, as it is the key stage for native Chinese children to learn to read independently and gradually become fluent readers (Cheung et al., 2009). It is a critical time period for the development of literacy for Chinese children, during which they extensively learn Chinese language and practice phonological skills in primary school (Shu et al., 2003).

In our study, we added longitudinal evidence on the development of lexical tone categorical perception. We try to bridge the predictive role of speech perception on reading. We chose the classical oddball paradigm and the stimuli chosen were very classical speech stimuli (Xi et al., 2010), a set of stimuli that has been used in many studies (Yu et al., 2014, 2017). The neural marker we selected was late MMN, and this component, together with early MMN, confirmed its sensitivity in groups such as autistic children and bilingual Cantonese and Mandarin (Yu et al., 2015, 2017). To measure children’s phonological awareness as well as their reading ability, we selected classic behavioral tasks that have been used in previous studies. The first one is the classical task of categorical perception, the identification task (Xi et al., 2010). This task contains only basic cognitive processes such as decision-making and involves fewer cognitive abilities and a purer description of perceptual abilities than other behavioral tasks. The second one is the tone detection task in Chinese that measures phonological awareness, which Chinese children are generally better able to perform after learning the rules of pinyin (Shu et al., 2006; Lei et al., 2011). The rest tasks are reading-related tasks, including Chinese Character Recognition (Li et al., 2012) as well as Reading Fluency (Landerl et al., 2009). Both tasks involve very complex advanced reading processes that involve a wealth of reading-related cognitive skills.

The current study aimed at determining the developmental change of pre-attentive cortical speech processing in Chinese children from the age 8 to age 10 that may underlie the CP of Mandarin lexical tones. Specifically, we investigated whether there is a more pronounced tone category effect on MMN from children at age 10 compared with that when they were at age 8, which may indicate the continuous development of lexical tone processing in Chinese children at school-ages. Furthermore, we try to find a connection between basic auditory processing and reading ability. We investigated how auditory brain responses recorded at the 2nd school grade is associated with later reading measures and cognitive skills known to be important for the development of literacy skills. we hypothesized that EEG response to the auditory stimuli might predict the later language-related abilities.

## Materials and methods

### Participants

Eighteen children participated in the experiment after having given informed consent, of whom 3 had to be excluded because of insufficient usable EEG data (one with curly hair; one was hyperactivity and one had a cough). The 15 children had a mean age of 99.27 months (S.D. 4.51 m, range 91–108 months, 7 male and 8 female) at the first test point. All were right-handed (handedness assessed using the Edinburgh Inventory) and reported having no neurological or hearing impairment. Parents were accompanying the children throughout the experiment to assist in the preparation of the experiment. The children were assessed both behaviorally and using EEG for two times. All participants were tested twice, with 2 years in between testing session. All of the children had Chinese as a first language. Parental informed written consent was obtained for all participants. The study received ethical approval from the ethical committee of Beijing Normal University.

### Stimuli

A native Chinese female speaker produced the two Chinese monosyllables /pa/ with tone2 and tone 4 (Tone2, the high rising tone, and Tone 4, the falling tone). These two sounds differed in their lexical tones. To meet these criteria, we used the method developed by Xi and colleagues which uses a continuum to determine equally spaced sounds between two different tones. They were digitally edited using Sound-Forge (SoundForge9; Sony Corporation, Tokyo, Japan), and were 200 ms in duration. The two stimuli were identical with each other except for the pitch contour difference. The /pa2/ and /pa4/ stimuli were taken as the endpoint stimuli and a morphing technique was then performed in MATLAB (MathWorks Corporation, Natick, MA, United States) using STRAIGHT (Kawahara et al., 1999) to create a 10-interval lexical tone continuum (Xi et al., 2010). All the 11 stimuli in the /pa2/−/pa4/ lexical tone continuum were used in the behavioral identification test. Based on the results from the adult behavioral test (Xi et al., 2010), three stimuli were chosen for the ERP oddball paradigm experiment, which are the third one, the seventh one, and the eleventh one of the 10-interval lexical tone continuum. In particular, the seventh one was used as the standard stimulus, and the third one (a between-category deviant) and 11th one (a within-category deviant) were used as two kinds of deviants. These two deviants have the same acoustic distance with the standard stimulus.

### ERP experimental procedures

The deviant stimuli 3(between-category deviant) and 11(within-category deviant) were presented in the same block against the standard stimulus 7(standard). In the oddball recording blocks, two rare deviants were presented quasi-randomly in the standard stream (Kujala et al., 2006; Naatanen et al., 2007), each with a probability of 0.1. Each participant completed one block containing 100 trials for each kind of deviant. The block was pseudo-randomized with a minimum of two standard stimuli occurring between two deviant stimuli. The stimulus onset asynchrony was 1,000 ms and stimuli were presented *via* a loudspeaker. In an acoustically and electrically shielded cabin, participants were instructed to watch short silent video films and to ignore auditory signals. The duration was approximately 18 min.

### EEG recording and data processing

The electrical signal (sample rate 500 Hz) was recorded during auditory stimulation using a 128-channel HydroCel Geodesic Sensor Net referenced to the vertex electrode (Tucker, 1993). Electrode impedances were kept below 50 kΩ. Data processing was carried out using the freeware EEGLAB toolbox for MATLAB (Delorme and Makeig, 2004). The signals were off-line down-sampled at 250 Hz, band-pass filtered at 0.3-30 Hz, and re-referenced to the common average. The EEG epochs, starting at 100 ms before stimulus onset and ending 600 ms after it, were averaged for each item and for each participant separately for standard and deviant stimuli. Trials containing ocular artifacts (monitored at electrodes below, above, and next to the eyes) and trials with voltage exceeding ± 120 μV at any of the recording electrodes were rejected from further analysis. The accepted minimum trial number was 80 per condition. The artifact-free standard trials which were closest before the deviant trials and all artifact-free deviant trials were used to calculate the mean ERP responses. The EEG data were corrected by a baseline of 100 ms before stimulus onset. Data from bad channels for each participant were interpolated (Perrin et al., 1987). The MMN response was obtained by subtracting the ERP response elicited by the standard stimuli from that elicited by the deviant stimuli.

### Behavior and outcome measures

#### Identification

After familiarization with the endpoints of the continuum, subjects were presented with trials that contained a single stimulus that could come randomly from anywhere in the continuum. They were then forced to choose which endpoint it belongs to. Subjects had 2 s to make their selections and, following each choice, were given 500 ms of silence prior to presentation of the next trial. Each of the 11 possible stimuli from the continuum was presented 10 times in a pseudo-random order.

#### Tone detection

Following the identification task, subjects performed a tone detection task. For each trial in this task, subjects heard three syllables, each separated from the next by 1,000 ms of silence. They must focus on the tone only. In this task, these syllables could be any tone of the four lexical tones. Subjects were asked to judge which syllable contains a different lexical tone from the other two. An example from the experiment would be a presentation of a hua1 (the first one), followed by a hong2 (the second one) and a kai1 (the third one). After each presentation, subjects were asked to click “1” on the keyboard if they believed the tone of the first syllable is different from the other two or to click “2” if they believed the second is different or to click “3” if they believed the third is different. In the above example, a response of 2 is correct. Following each response, there was a 2 s silent period prior to the next trial. Subjects were not provided with feedback about their accuracy.

#### Chinese literacy tasks

Based on previous research (Shu et al., 2006; Lei et al., 2011), here are another two tasks chosen to measure Chinese literacy. These tasks represent several cognitive abilities, which are very important for Chinese reading development.

##### Chinese character recognition

This task was used in our previous studies. One hundred and fifty single-character words were arranged in order of increasing difficulty (Li et al., 2012). This task is applicable to children at different grade levels. It has a broad band of usage frequency and covers different types of characters according to the distributions in the primary textbook (42 are regular characters, 62 are irregular characters, and 46 are not phonogram). During the task, the experimenter faced the child across the table and presented a stimuli book. Each child was asked to read each character aloud at his/her own pace; in the meanwhile, the experimenter marked on the answer sheet and stopped the child once he/she failed 15 consecutive items. A score of one was assigned to each correctly pronounced item and the maximum score was 150.

##### Reading fluency

Reading Fluency task is similar with the Woodcock-Johnson 3-Tests of Achievement (Woodcock et al., 2001). It was a timed task, consisting of 100 items with sentences listed line by line on A4 pages (Landerl et al., 2009). The sentences were arranged in the order of increasing number of characters. Children were given 3 min to read in silence and make judgments on the truth of each sentence (e.g., “大象比蚂蚁小” The elephant is smaller than the ant). All the items were written in easy, familiar characters, and children were told to do it as fast and accurate as possible. The sum of the characters of the correctly judged sentences was counted for each individual. The maximum score is 3,927.

#### Behavior data analysis

In order to derive a common factor that is based on the theoretical construct defined in the literature, a principal component analysis (PCA) with Varimax rotation was conducted. This allows us to examine the interrelations among different dependent variables and identify the underlying structure of these variables. The case-to-variable ratio in this study was 9:1 (36:4). The Kaiser–Meyer–Olkin measure of sampling adequacy confirmed the validity of using a factor analysis for structure detection. The different reading processing domains were well characterized using a two-factor solution. The two primary PCA factors (Eigenvalues: 2.013 and 1.270) collectively contributed to 82% of the overall variance. In order to interpret the contribution of each variable toward a factor, component loadings greater than a value of 0.5 were considered significant and designated according to the main construct captured. The current study identifies two common factors, labeled as readflu and tonecat, that are associated with reading fluency and categorical tone perception, respectively.

## Results

Prior to data analysis, each of the data sets was checked for outliers on linear regression. Any data point that had a Cook’s distance score > 4/N (0.2667) was removed in order to prevent that point having undue influence on the results. We then ran the analyses based on the new subset of participants.

**Figure 1:**
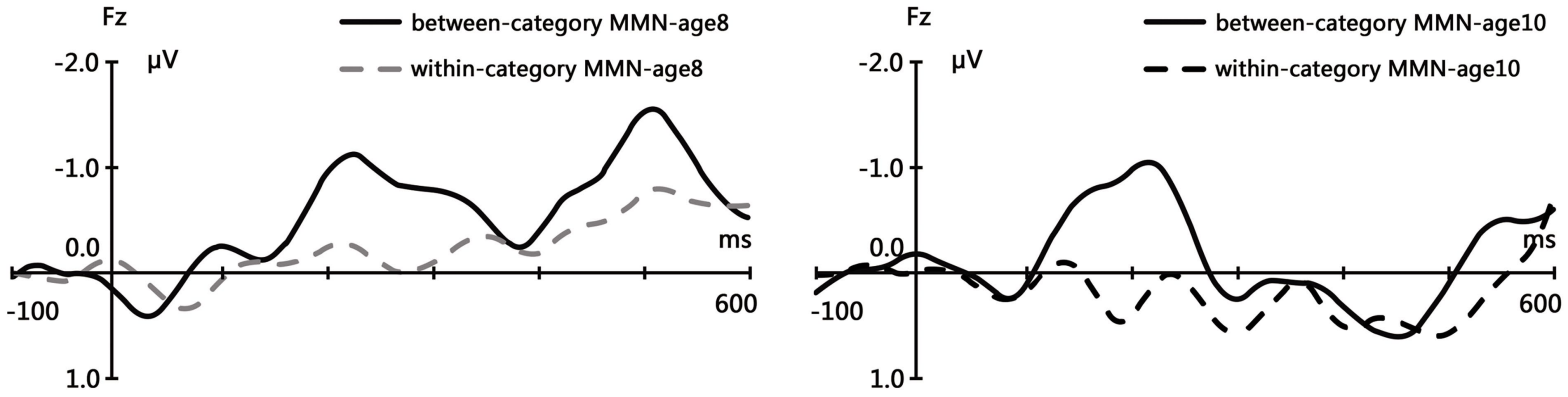
Grand average waveforms of Mismatch negativity (MMN) responses elicited by the between-category deviants (*p* = 10%) and within-category deviants (*p* = 10%) from the Fz electrode location.

The grand average waveforms for within-category MMN and between-category MMN are shown in Figure 1. Early and Late negative peaks were observed in the deviant-minus-standard difference waves for both time points (the first time point age 8 and 2 years later) at the electrode location Fz. The mean amplitude of early MMN was extracted from 180 to 240 ms and the late MMN component was extracted from 470 to 540 ms according to the grand average waveforms. For the first time point, one-sample *t*-test demonstrated that the early MMN component was significantly present at Fz electrode in between condition but in the within condition, *t* = −3.217, *p* = 0.006; *t* = −0.076, *p* = 0.940. The LMMN component significantly showed out on both conditions (Between-category and Within-category), *t* = −5.800, *p* < 0.001; *t* = −2.533, *p* = 0.024. For the second time point, the early MMN component was significantly present at Fz electrode in between condition but in the within condition, *t* = −2.366, *p* = 0.033; *t* = 1.070, *p* = 0.303. There is not a significant component for any condition for late MMN (*t* = 0.674, *p* = 0.511; *t* = 1.011, *p* = 0.329).

**Figure 2:**
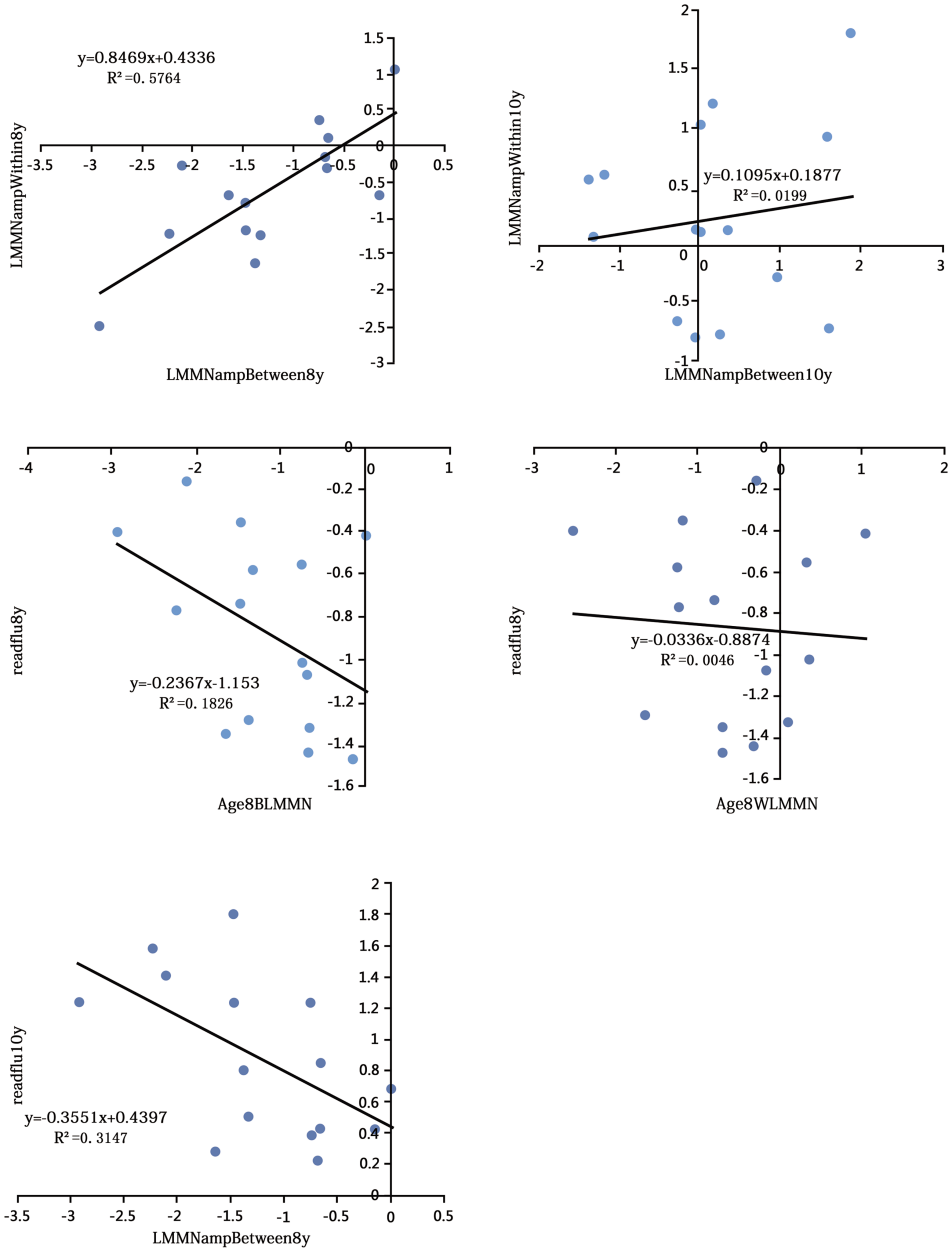
Scatter plots display the relationship between MMN amplitude values and reading ability.

The scatter plots display the relationship between MMN amplitude and reading ability in Figure 2. Correlations between EEG and cognitive skills were done. Pearson’s correlation analyses (see Table 1) revealed that, for the first time point, the late MMN components for both conditions are significantly correlate with each other (*r* = 0.759, *p* = 0.001). However, they become not significant at age 10 (*r* = 0.141, *p* = 0.616). When they are age 8, the LMMN for between-category correlate with their concurrent readflu score (*r* = −0.427, *p* = 0.112; Cook’s D adjusted *r* = −0.625, *p* = 0.017), but LMMN for within-category does not (*r* = 0.068, *p* = 0.81). Between-category LMMN at age 8 can predict children’s readflu score at 2 years later (*r* = −0.561, *p* = 0.03). The regression model with Between-category LMMN at age 8 as the independent variable and readflu score at age 10 as the dependent variable is significant (*b* = −0.561, *t* = −2.443, *p* = 0.03). For early MMN, there was no significant correlation between early MMN and any behavior score (see Table 1).

**Table 1:** Correlation coefficients between EEG and cognitive skills.

	1	2	3	4	5	6	7	8	9	10	11
1. readflu8y	1										
2. tonecat8y	−0.179	1									
3. readflu10y	0.601^*^	0.237	1								
4. tonecat10y	−0.272	0.344	−0.027	1							
5. Age8BMMN	−0.014	−0.184	−0.211	−0.289	1						
6. Age8WMMN	−0.011	−0.133	−0.211	−0.468	0.869^**^	1					
7. Age10BMMN	−0.084	−0.179	−0.165	0.046	0.151	0.128	1				
8. Age10WMMN	−0.047	−0.433	−0.202	0.201	0.26	−0.068	0.43	1			
9. LMMNampBetween8y	−0.427	0.255	−0.561^*^	−0.125	0.054	0.135	−0.093	−0.067	1		
10. LMMNampWithin8y	−0.068	0.202	−0.284	−0.278	−0.414	−0.165	−0.314	−0.435	0.759^**^	1	
11. LMMNampBetween10y	0.298	−0.134	−0.043	−0.071	−0.187	−0.085	0.487	0.41	0.236	0.263	1
12. LMMNampWithin10y	0.321	−0.396	0.156	0.051	0.176	0.101	−0.248	0.249	0.017	−0.064	0.141

** *p* < 0.01 (2-tailed);

* *p* < 0.05 (2-tailed).

LMMNampBetween8y and readflu8y’s correlation showed out after we remove one Cook’s D outliers (*r* = −0.625, *p* = 0.017); LMMNampWithin10y and readflu10y’s correlation also became significant after we remove two Cook’s D outliers (*r* = 0.760, *p* = 0.003).

## Discussion

Categorical perception plays a critical role in children’s reading development (Liu and Tsao, 2017). Although dyslexia is now thought to be a multifactorial disorder, many research have demonstrated that speech perception in dyslexia is characterized by a reliable categorical perception deficit (Godfrey et al., 1981; Werker and Tees, 1987; Noordenbos and Serniclaes, 2015). This study attempts to explore the predictive role of lexical tone categorical perception on children’s reading ability. Two behavioral tasks of phonological awareness (tone awareness factor) and two behavioral tasks of reading (reading factor) are used, and the cortical responses of categorical perceptual sensation to lexical tone are analyzed to explore the predictive effect of brain on behavior. Late MMN component was selected as an indicator of brain level indicator (Table 2).

**Table 2:** Descriptive statistics for behavioral measures and MMN.

	*N*	Minimum	Maximum	Mean	Std. deviation
readflu8y	15	−1.4778	−0.1651	−0.8677	0.4458
tonecat8y	15	−1.0703	1.1022	−0.0398	0.7119
readflu10y	15	0.2198	1.7955	0.8677	0.5094
tonecat10y	15	−1.4818	3.4302	0.0398	1.2495
LMMNampBetween8y	15	−2.9151	0.0150	−1.2053	0.8048
LMMNampWithin8y	15	−2.5039	1.0517	−0.5872	0.8977
LMMNampBetween10y	15	−1.3818	1.8889	0.1780	1.0222
LMMNampWithin10y	15	−0.8082	1.7933	0.2072	0.7940

The results of the correlation analysis show that the late MMN component of between-category significantly correlated with within-category at the age of 8. However, by the age of 10, the correlation disappeared. At the age of 8, the late MMN component of between-category was associated with the reading factor at the time, but the late MMN of within-category was not. At the age of 8, children’s late MMN of between-category can predict their reading ability at 10-year-old.

We found a change of the relationship between between-category and within-category perception from age 8 to age 10. This change reflects the development of CP. A study with Australian children suggested that the perception of allophonic contrasts was negatively related to school experience (Horlyck et al., 2012). The effect of school experience on allophonic sensitivity was confirmed in a longitudinal study with Dutch children at risk for dyslexia. These children exhibited a strong CP deficit when they were in Kindergarten; however, it completely disappeared when they were in the first grade (Noordenbos et al., 2012). It seems that allophonic perception is not specific to dyslexia and it decreases with reading experience. Our research is consistent with these findings. The CP is still developing after school experience. Children treat between- and within-category similar at age 8 which shows a stronger allophonic perception. The phonemic discrimination on between-category no longer correlates with within-category perception at age 10.

We found a directly relationship between CP and reading. The between-category MMN is significantly related with reading factor at age 8. Dyslexia is often ascribed to an underlying deficit in phonemic awareness, it may be caused by a more remote deficit in the perception of phoneme categories which can either lead to a deficit in phonemic awareness and thus in turn to sound-letter matching difficulties (Noordenbos and Serniclaes, 2015). The most common manifestation of the CP deficit in dyslexia is weaker discrimination of acoustic differences between phonemic categories in conjunction with better discrimination of acoustic differences within phonemic categories (Werker and Tees, 1987; Serniclaes et al., 2001). Allophonic perception should be reorganized during the 1^st^ year of life according to the contrasts present in the ambient language (Hoonhorst et al., 2009). School experience might enhance the use of top-down strategies to focus on relevant contrasts and ignore irrelevant ones (Horlyck et al., 2012). Therefore, allophonic perception blurs the relationships between phonemes and graphemes and highly disrupts the reading acquisition of dyslexia.

Interestingly, this study is a companion to a paper published on JCPP in 2012. In the study of Zhang et al., we found that there is a difference between dyslexia and control group in categorization perception of MMN. 10-year-old children with dyslexia are oversensitive to stimuli within-category. The development of children’s CP is reflected in the decrease of MMN amplitudes within-category and no change on MMN amplitudes between categories, resulting in differences between two conditions. The 8-year-old children in this study are the reading level control group of Zhang et al. Their MMN components are different from those of 10-year-old dyslexic children, indicating that the CP of 10-year-old dyslexic children is a variation of development rather than a lag of development.

In order to answer whether the relationship between CP and reading is accompanying or causal, longitudinal research has become the most ideal experimental design for researchers. In the past, research on predicting children’s behavior through the electrophysiological index of MMN is very rare. Only a few studies are concerned with the prediction of event-related potential of infants or preschoolers to reading ability in childhood. Shortly after the baby is born, the brain’s response to auditory stimuli can predict subsequent reading-related cognitive abilities (Maurer et al., 2009; Leppänen et al., 2010; Van Zuijen et al., 2012; Plakas et al., 2013; Schaadt et al., 2015). Children with dyslexia risk are significantly different from those of the control group and the brain level indicator can predict children’s future reading difficulties. A study by Tallal (2004) found that children’s ability to process rapid input of auditory information can predict the ability to decode fast-present, meaningless speech stimuli. This study is consistent with previous studies (Tallal et al., 1993; Tallal, 2004). It also found that the underlying brain mechanism of the categorization of lexical tone in 8-year-old children has a significant predictive effect on children’s reading factors after 2 years.

## Conclusion

The ability to categorize lexical tone is still developing from age 8 to age 10. Speech perception development is a protracted process in which children increasingly sharp phonetic categories. Categorical perception of lexical tone at age 8 predicts children’s reading ability at age 10 in normal Chinese children. This study did not include data from children with dyslexia. In future studies, the late MMN characteristics of dyslexic children can be further explored. And a comparison between pre-and post-intervention of dyslexia could be conducted. These research will eventually help us to gain a more complete and in-depth understanding of the relationship between categorical perception, phonological awareness, and reading ability.

## Data availability statement

The raw data supporting the conclusions of this article will be made available by the authors, without undue reservation.

## Ethics statement

The studies involving human participants were reviewed and approved by the Ethical Committee of the Beijing Normal University. Written informed consent to participate in this study was provided by the participants’ legal guardian/next of kin.

## Author contributions

HW: conceptualization, methodology, formal analysis, data curation, and original draft writing and editing. YZ: visualization, software, and manuscript revision. All authors contributed to the article and approved the submitted version.

## Funding

This research was supported in part by the grants from National Social Sciences Fund of China (NSSFC, 2015 Major Project), A Study on the Standard and Assessment System of Communicative Ability, project no. 15 ZDB 101 and the Science Foundation of Beijing Language and Cultural University (supported by “the Fundamental Research Funds for the Central Universities”) (17YBB22 and 21PT02) and the Discipline Team Support Program of Beijing Language and Culture University, JC201903, and BLCU Youth Talent Development Program.

## Conflict of interest

The authors declare that the research was conducted in the absence of any commercial or financial relationships that could be construed as a potential conflict of interest.

## Publisher’s note

All claims expressed in this article are solely those of the authors and do not necessarily represent those of their affiliated organizations, or those of the publisher, the editors and the reviewers. Any product that may be evaluated in this article, or claim that may be made by its manufacturer, is not guaranteed or endorsed by the publisher.
