# Supplementary figures and images for: Late mismatch negativity of lexical tone at age 8 predicts Chinese children’s reading ability at age 10
Source: Front Psychol. 2022 Oct 21;13:989186. doi: 10.3389/fpsyg.2022.989186 (PMC9633667; doi:10.3389/fpsyg.2022.989186)

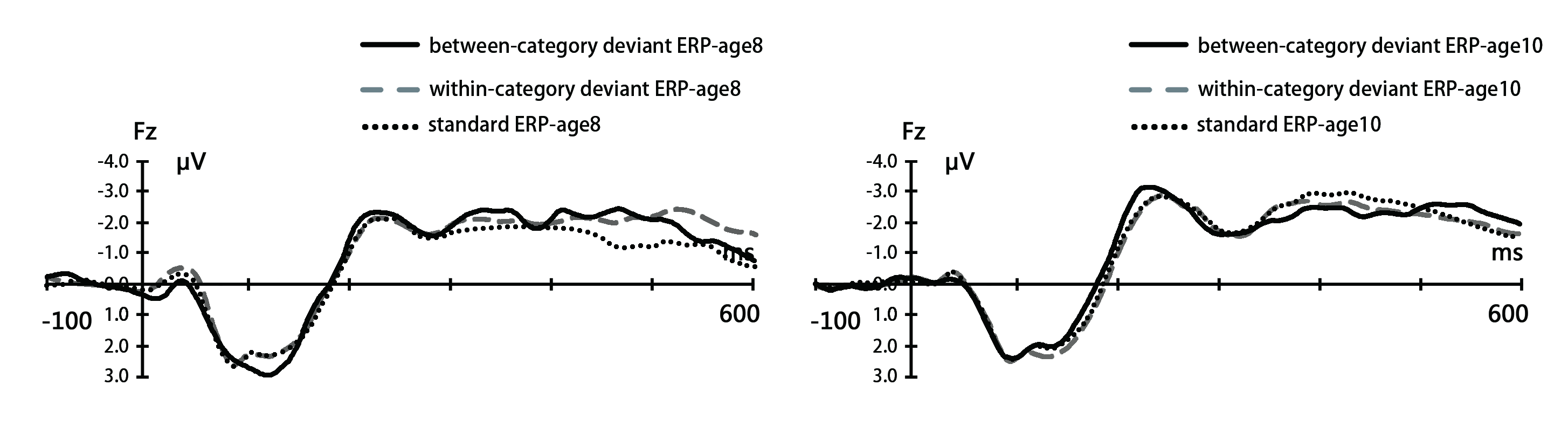

Supplement: Supplementary file 1 [file Image_1.TIF]
